# Corrigendum to Zha (2019), Transforming brain signals related to value evaluation and self‐control into behavioural choices

**DOI:** 10.1002/hbm.25113

**Published:** 2020-07-16

In the above‐mentioned article, we reported a study in which participation was limited to men (Zha, Bu et al. 2019). The justification to it was:

***Deletions:***

**Methods**

“To avoid intense emotions effect on the decision making (Al Omari, Razeq et al. 2016, Lempert and Phelps 2016), females were not recruited in the present study.”

Limitations:

“Second, only male participants were recruited in the present study; we did not focus on the gender effect on the decoding results. Previous studies have shown that intense emotions are common symptoms in females during menstruation (Al Omari et al. 2016; Bata, 2012) and emotions affect intertemporal choices (Lempert and Phelps 2016). Therefore, we did not recruit female participants for the present study. Whether the findings can be generalized to female participants remains to be demonstrated in future investigations.”

***Reasons for deletions:***

The reference we provided cannot be reasonably construed as justifying the limitation of study participants to males. Therefore, the authors deleted the sentences listed above in the method section and limitation section. Al Omari et al (2016) have suggested menarche in adolescent girls. Bata (2012) have shown that there were profound negative emotions during the menstruation. Bata (2012) have also shown that one‐third of female students showed irregular cycles, therefore, the menstruation would be difficult to predict and be controlled during experiment. Lempert and Phelps (2016) have reviewed that the negative emotions would affect intertemporal choices. Ossewaarde, van Wingen et al. (2013) have found emotion regulation and differences in brain activation in the menstrual cycle under stress induction, suggesting that menstrual cycle modulates brain activity. Therefore, we inferred that female during the menstruation would show altered intertemporal choices. However, no studies have shown female menstruation affect intertemporal choice or other decision‐making contexts. Furthermore, we did not discuss menarche in the discussion section. Therefore, it is not appropriate to use an inference as a recruiting criterion in the methods and limitation sections.

***Additions:***

Limitations:

“Second, only male participants were recruited in the present study. The relative incidences of both addictions differs in China (Tao, Huang et al. 2010, Wang, Lyu et al. 2015), with men being highly susceptible to smokers/Internet gaming disorder and woman being rarely affected. When we commenced recruitment of participants, we did not restrict the gender, but after recruiting more than 20 male smokers/Internet gaming disorder and only one female smoker, we decided to modify the study protocol to be a single gender study. Mavrogiorgou, Enzi et al. (2017) have shown that superior frontal gyrus signal difference between immediate reward and delayed reward was correlated with the loudness dependence of auditory evoked potentials only for female participants, but not for male participants. These results suggest differential activations between female and male during the intertemporal choices. Therefore, whether the conclusion in the present study could be generalized to female should be investigated in future work. The field would also benefit from diverse ethnic in future work.”

We have requested approval from the Human Research Ethics Committee of the University of Science and Technology of China to amend the study design to exclude females from the recruitment protocol. The final study protocol was compliant with the Human Research Ethics Committee of the University of Science and Technology of China requirements.

Al Omari, O., N. M. A. Razeq and M. M. Fooladi (2016). “Experience of menarche among Jordanian adolescent girls: An interpretive phenomenological analysis.” Journal of pediatric and adolescent gynecology **29**(3): 246–251.

Bata, M. S. (2012). “Age at menarche, menstrual patterns, and menstrual characteristics in Jordanian adolescent girls.” International Journal of Gynecology & Obstetrics **119**(3): 281–283.

Lempert, K. M. and E. A. Phelps (2016). “The Malleability of Intertemporal Choice.” Trends Cogn Sci **20**(1): 64–74.

Mavrogiorgou, P., B. Enzi, A. K. Klimm, E. Kohler, P. Roser, C. Norra and G. Juckel (2017). “Serotonergic modulation of orbitofrontal activity and its relevance for decision making and impulsivity.” Hum Brain Mapp **38**(3): 1507–1,517.

Ossewaarde, L., G. A. van Wingen, M. Rijpkema, T. Backstrom, E. J. Hermans and G. Fernandez (2013). “Menstrual cycle‐related changes in amygdala morphology are associated with changes in stress sensitivity.” Hum Brain Mapp **34**(5): 1187–1,193.

Tao, R., X. Huang, J. Wang, H. Zhang, Y. Zhang and M. Li (2010). “Proposed diagnostic criteria for internet addiction.” Addiction **105**(3): 556–564.

Wang, X., J. Lyu, Y. Guo, Z. Bian, C. Yu, H. Zhou, Y. Tan, P. Pei, J. Chen, Z. Chen, L. Li and G. China Kadoor Biobank Collaborative (2015). “Regional differences in adults’ smoking pattern: findings from China Kadoorie Biobank study in 10 areas in China.” Chin J Epidemiol **36**(11): 1200–1,204.

Zha, R., J. Bu, Z. Wei, L. Han, P. Zhang, J. Ren, J.‐A. Li, Y. Wang, L. Yang, S. Vollstadt‐Klein and X. Zhang (2019). “Transforming brain signals related to value evaluation and self‐control into behavioral choices.” Human brain mapping **40**(4): 1049–1,061.

